# Objective analysis of partial three-dimensional rotator cuff muscle volume and fat infiltration across ages and sex from clinical MRI scans

**DOI:** 10.1038/s41598-023-41599-z

**Published:** 2023-09-01

**Authors:** Lara Riem, Silvia S. Blemker, Olivia DuCharme, Elizabeth B. Leitch, Matthew Cousins, Ivan J. Antosh, Mikalyn Defoor, Andrew J. Sheean, Brian C. Werner

**Affiliations:** 1 Springbok Analytics, Charlottesville, VA USA; 2 University of Virginia Medical School, Charlottesville, VA USA; 3 San Antonio Military Medical Center, San Antonio, TX USA

**Keywords:** Three-dimensional imaging, Skeletal muscle

## Abstract

Objective analysis of rotator cuff (RC) atrophy and fatty infiltration (FI) from clinical MRI is limited by qualitative measures and variation in scapular coverage. The goals of this study were to: develop/evaluate a method to quantify RC muscle size, atrophy, and FI from clinical MRIs (with typical lateral only coverage) and then quantify the effects of age and sex on RC muscle. To develop the method, 47 full scapula coverage CTs with matching clinical MRIs were used to: correct for variation in scan capture, and ensure impactful information of the RC is measured. Utilizing this methodology and automated artificial intelligence, 170 healthy clinical shoulder MRIs of varying age and sex were segmented, and each RC muscle’s size, relative contribution, and FI as a function of scapula location were quantified. A two-way ANOVA was used to examine the effect of age and sex on RC musculature. The analysis revealed significant (p < 0.05): decreases in size of the supraspinatus, teres minor, and subscapularis with age; decreased supraspinatus and increased infraspinatus relative contribution with age; and increased FI in the infraspinatus with age and in females. This study demonstrated that clinically obtained MRIs can be utilized for automatic 3D analysis of the RC. This method is not susceptible to coverage variation or patient size. Application of methodology in a healthy population revealed differences in RC musculature across ages and FI level between sexes. This large database can be used to reference expected muscle characteristics as a function of scapula location and could eventually be used in conjunction with the proposed methodology for analysis in patient populations.

## Introduction

Rotator cuff (RC) tears remain a challenging clinical problem, causing functional impairments including pain, limited active range of motion, and weakness. RC repairs are the second most common orthopedic soft tissue surgery^1^, with over 250,000 performed annually in the United States^2^. Approximately 20% to 50% of individuals over 60 years of age have a known RC tear^3,4^. Despite clinical pervasiveness and the evolution of surgical techniques, outcomes of RC repair remain highly variable, particularly for larger, retracted, and chronic tears. Muscle atrophy and fat infiltration of the RC muscles play significant roles in determining healing and functional outcome after repair^5^. Current assessments of RC musculature rely on the subjective grading of a singular computed tomography (CT) or magnetic resonance image (MRI). The most common of these methods include the Goutallier score^6^, Fuchs score^7^, tangent sign^8^, and occupation ratio^9^. The Goutallier score aims to qualitatively assess the muscles from stage 0 (normal muscle with absence of fat infiltration/replacement) to stage 4 (more fat infiltration/replacement than muscle). Occupation ratio, a more quantitative approach, relates muscle, infiltrated fat, and the scapula fossa cross sectional area (CSA) to characterize muscle size and infiltration. However, all these methods have multiple limitations: (1) subjectivity and lack of precision, (2) limited applicability of 2D measurements to the entire muscle structure^10^, (3) bias towards the supraspinatus at the expense of the other RC muscles; and (4) lack of concrete normative comparisons to account for natural atrophy with age^11^. Current innovations have trended toward complete 3D preoperative planning^12^ and objective 3D fat quantification^13^; however, clinical MRI scans often only capture the lateral portion of the RC muscle structure. To systematically determine 3D muscle volume, a method to account for variation in capture range that is typical of clinical MRI scans must be developed and validated.

The goals of this study were (i) develop and validate a novel volumetric method of quantifying the morphology of all four rotator cuff muscles, their respective infiltrated fat, and the surrounding bones from clinically obtained MRI scans with the typical limited lateral coverage range and (ii) use the method to analyze healthy RC clinical MRI scans of varying ages and across sexes to examine sex- and age-related difference in RC muscle size and fat infiltration. We hypothesize that RC muscles exhibit increased atrophy and FI with age. The purpose of validating this study’s methodology and establishing its results in a healthy control population of varying demographics is for its eventual application in patient or athlete populations.

## Methods

### Overview of approach

**Figure 1:**
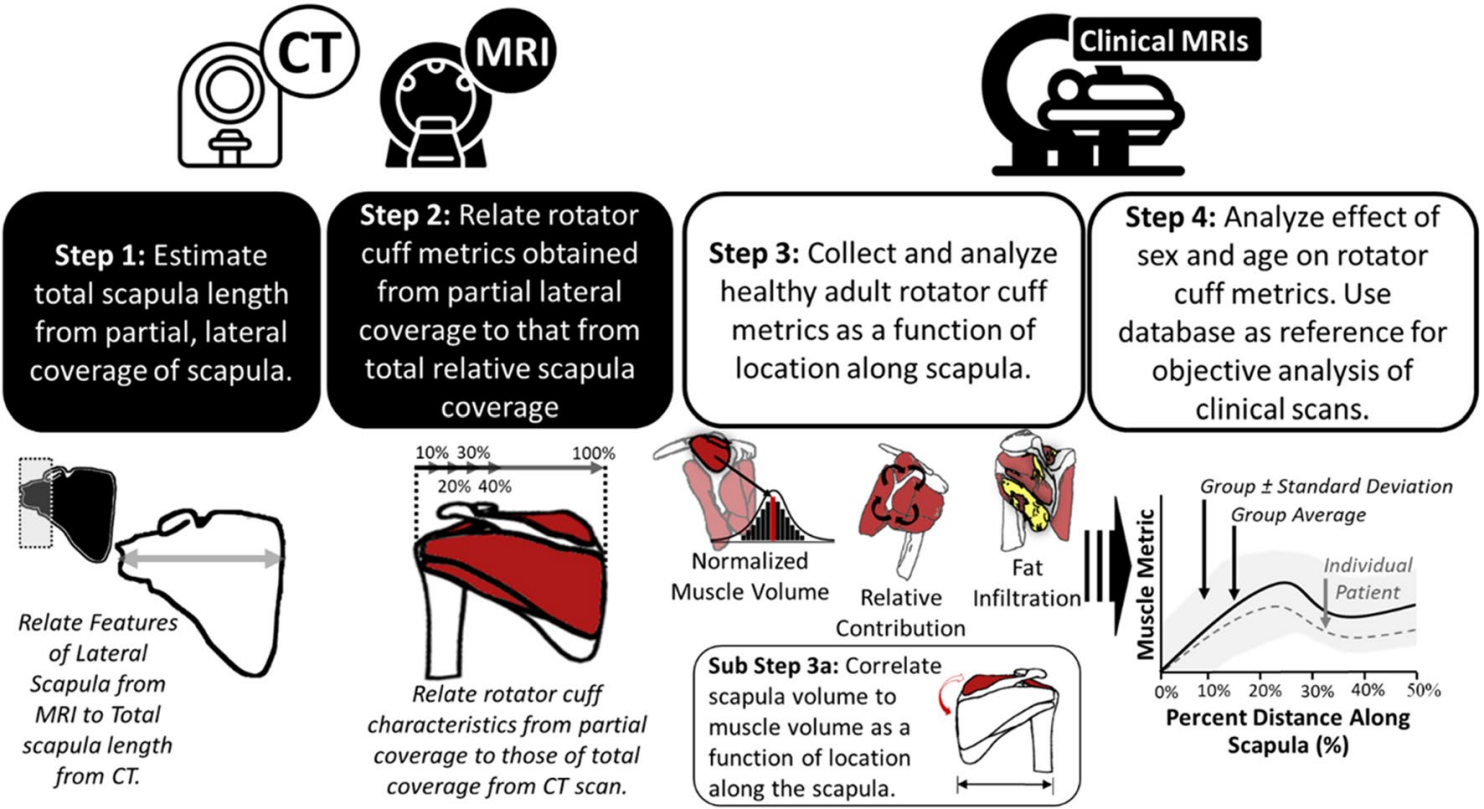
Overview of the paper’s methodology workflow and steps used for this paper. The process of analyzing and interpreting clinical rotator cuff MRI scans in this paper are summarized in four steps.

Development of the method to quantify muscle characteristics from clinically obtained sagittal RC MRI scans with partial coverage involved four main steps (Fig. 1). The first step concerned the creation and validation of a method to estimate total scapula length from partially captured scapula volumes. This would therefore allow the RC MRI scan capture range to be represented as a percentage of scapula captured and allow for normalization and interpretation between scans of varying coverage. In the second step, RC muscle characteristics obtained at varying partial capture ranges (typically measured in clinical MRI scans) were compared to their respective full coverage RC muscle characteristics. This analysis allowed us to determine if muscle metrics achieved from partial coverage would be as useful as full coverage metrics when understanding muscle morphology. Third, a previously developed AI-based approach^14^ was used to segment the RC muscles and fat quickly and accurately in a large healthy adult database of 170 clinical RC MRI scans of varying ages and sex. Metrics of each RC muscle’s size, relative contribution, and fat infiltration as a function of scapula coverage were quantified using step 1, and metrics measured from at least 30–40% scapula coverage provided reliable information based on step 2. Further, as a sub step, muscle volumes were normalized by scapula volume to account for variance in patient size. To ensure a strong relationship and validate the scapula’s use as a normalization technique in partially captured RC muscles, the RC muscle volumes were correlated to scapula volume at varying locations along the scapula. Finally, we examined the effects of age and sex on all RC muscle characteristics at 30% scapula coverage.

### Dataset

The entire dataset used for this study consisted of 170 shoulder MRI scans and 47 shoulder CT scans with matching MRI scans obtained retrospectively from one surgical clinic (Table 1). All experimental protocols were approved by the Institutional Review Board for Health Sciences Research at the University of Virginia, and all methods were carried out in accordance with relevant guidelines and regulations. As this study was a retrospective analysis, it met the criteria for an exempt determination and consent was not required by the Institutional Review Board for Health Sciences Research at the University of Virginia. All data was de-identified and contained the minimum necessary PHI before utilized in the study. All MRI scans were collected with a sagittal plane protocol used in typical clinical workflows. For MRI scans, this typical clinical workflow consisted of scans with fat suppression off (or a similar method in which infiltrated fat is visualized distinctly from the muscle), and resolution of at least 6 mm in the sagittal plane and 1 mm in-plane to ensure a consistent and accurate method of FI quantification and volume resolution for eventual musculature analysis. The datasets were separated into two groups: (1) complete coverage CT group (n = 47, patients with no injury to the scapula and had complete coverage CT scans of the scapula taken within 3 years of a matched clinical MRI), (2) a healthy rotator cuff group (n = 170, no diagnosed RC pathology confirmed by MRI inspection and clinician notes). Patients in the healthy RC group were scanned as an initial screening for upper body pain, in which the RC musculature was found to not be impacted (eventual capsulorrhaphy, biceps tendonesis, clavicle resection, etc.). In order to examine the differences in muscle characteristics across age and sexes, subgroups were created: for each biologic sex (male, female) and six age subgroups (15–29, 30–39, 40–49, 50–59, 60–69, 70–79 years).

**Table 1:** Demographics of the 47 CT/MRI patients and the 170 control patients. (A) Patient demographics for the paired MRI and CT RC scans used for the training and testing of CT scapula length prediction from distal MRI scapula morphology in the training and test group. Shown is the scan count (n), average age ± 1 standard deviation (years), sex break down, shoulder side, and the average and range of scapula length. The age was taken at the time of their MRI scans, in which the CT scan was taken within 3 years of the MRI. (B) The demographics for the control patients’ scans that make up the control database. Shown is the breakdown of scans by sex and age subgroup. The scan count and the average age ± 1 standard deviation (years) of the patients in each subgroup.

(A) Scapula length validation—matched CT/MRI scans						
	Age (years)	Sex	RC laterality	Scapula length (cm)		
Training group (n = 33)	38.57 ± 22.73	13 F, 20 M	14 L, 19 R	Average: 14.59 ± 1.73 cm Range: 11.63–19.80 cm		
Testing group (n = 14)	59.50 ± 16.24	5 F, 9 M	5 L, 9 R	Average: 14.76 ± 1.74 cm Range: 12.21–19.16 cm		

(B) Control patient scans						
	15–29 years	30–39 years	40–49 years	50–59 years	60–69 years	70–89 years
Male	*n* = 14 Average age: 22.1 ± 4.2	*n* = 14 Average age: 33.6 ± 2.6	*n* = 12 Average age: 45.0 ± 2.9	*n* = 15 Average age: 54.1 ± 3.2	*n* = 15 Average age: 63.9 ± 2.1	*n* = 15 Average age: 76.3 ± 5.0
Female	*n* = 12 Average age: 21.9 ± 5.1	*n* = 15 Average age: 34.5 ± 2.8	*n* = 13 Average age: 45.5 ± 3.0	*n* = 17 Average age: 54.7 ± 2.9	*n* = 13 Average age: 64.0 ± 3.0	*n* = 15 Average age: 72.8 ± 2.1

### Processing complete coverage CT and matched clinical MRI scans

**Figure 2:**
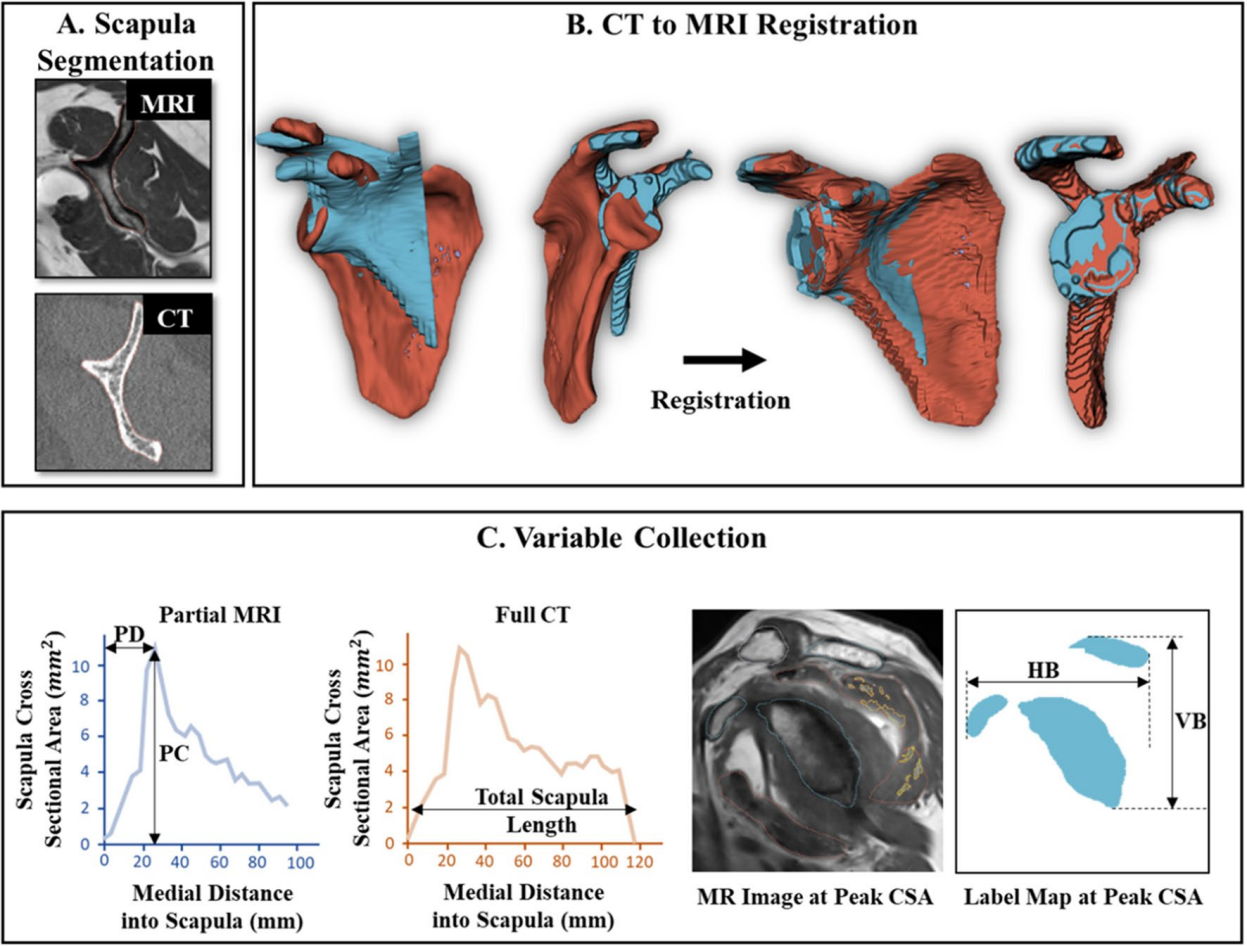
(**A**) Example segmentation of the scapula on a MRI (top) and matching CT (bottom) scan. (**B**) Registering the full CT (red) to the original partial MRI (blue) 3D rendering of the scapula to get final label maps. (**C**) Measuring the points used to relate the partial MRI scan’s scapula to the total scapula length from the CT scan. (Left) The scapula’s CSA as a function of its sagittal length for the MRI and CT scan. (Right) the scapula morphology at the point of peak scapula CSA. Sagittal distance of peak CSA (PD), CSA at point (PC), vertical (VB) and horizontal (HB) bounding length of the scapula at this point, and total scapula length.

In the complete coverage CT group (n = 47), segmentation of the scapula was performed by trained segmentation engineers on both the MRI and respective CT scan for each patient, producing a 3D volume rendering of the scapula from each scan (Fig. 2A). To account for different scan plane orientations, the labeled scapula from the CT scan was registered in 3D to the labeled scapula from the MRI. Registration was performed by zero padding the MRI and using Advanced Normalization Tools (ANTs) to complete a rigid 3D registration^15^. This algorithm transforms the CT data to the MRI data via translation and rotation (no scaling or shearing) to produce the least amount of difference between the two 3D label maps (Fig. 2B).

### Development of a method to predict scapula length from lateral morphology

The full CT and partial MRI scans used for the following analysis were derived from a cohort of patients with no injuries to their scapula. The method to predict scapula length from a partial scan involved analyzing multiple features within and relative to the image that contained the peak CSA of the scapula. We chose this point because it provided a location that was consistently identifiable for all scans. Four measures were taken (Fig. 2C): the (1) sagittal distance from peak CSA to most lateral point on scapula (PD), (2) peak CSA (PC), (3) vertical dimension of the bounding box (VB) of the scapula, and (4) horizontal dimension of the bounding box (HB) of the scapula. The peak CSA of the scapula was only found looking within a window of the first half of the scapula, it is possible there is another peak in scapula CSA as it continues moving medially, but as we are dealing with clinical MRIs with limited lateral coverage, that would be an unneeded landmark. To establish how the variables relate to total scapula length along the sagittal plane, the paired MRI and CT scans were used. The MRI scan was used to determine the four measures listed above, and the CT scan was used to calculate total length of the scapula (measurements from the MRI were checked against those obtained from the CT for consistency, all differences were < 5% on average). While it is possible there could be some change in scapula structure given the MRI and CT scans were administered at differing timepoints, this is unlikely as the inclusion criteria restricted the difference in scan date to 3 years.

A multivariable linear regression was used to relate the four MRI measures to the total length of the scapula obtained by the CT scan for 33 of the patients (randomly selected). All variables were normally distributed as discerned by Shapiro-Wilks test (p > 0.05). To evaluate the output equation, the predicted scapula length of 14 of the scans that were not included in the initial regression were compared with the length predicted by the regression.

### Processing healthy rotator cuff MRI scans

**Figure 3:**
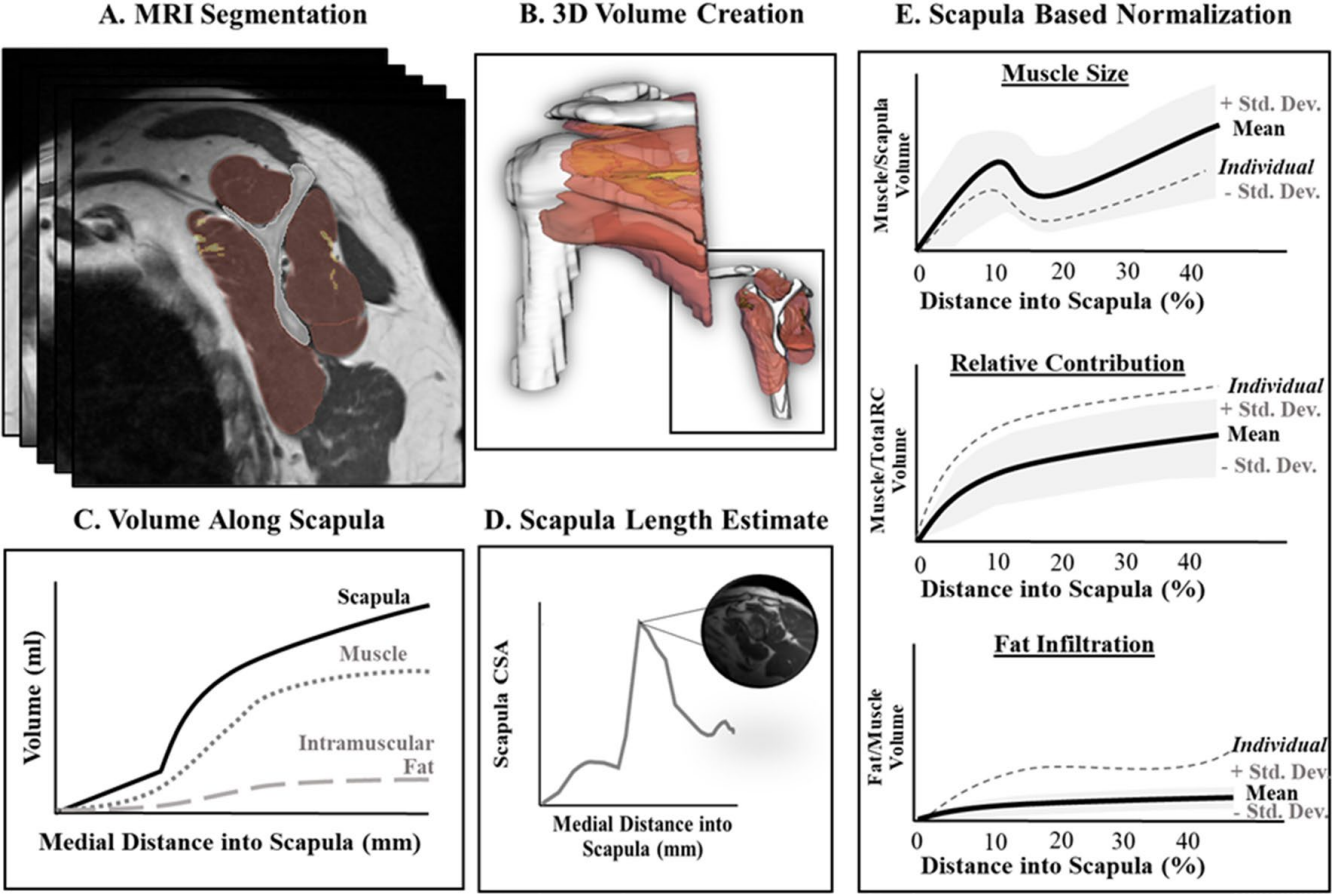
Visual overview of the rotator cuff processing. (**A**) Segmenting the RoIs from the MRI scan. (**B**) 3D-volume rendering of the RoI segmentation. (**C**) Cumulative volume (ml) of the scapula (solid line), a muscle (dotted line), and intramuscular fat (dashed line) RoI as a function of distance along the scapula (mm) moving medially. (**D**) Predicting the total scapula length (mm) from morphological features of the lateral scapula. (**E**) The cumulative muscle volume normalized by the scapula volume and presented as a function of percent distant into scapula (%) for the muscle size, relative contribution, and fat infiltration volumetric score.

Segmentation of the RC musculature, fatty infiltration, and bones from the 170 clinical MRI scans was performed automatically using 3D artificially intelligent segmentation^14^ and was vetted to ensure accuracy by three trained segmentation engineers. 2D label maps (Fig. 3A) and 3D volume renderings (Fig. 3B) were generated of the following regions of interest (RoIs): (1) humerus, (2) scapula, (3) clavicle, (4) supraspinatus, (5) supraspinatus fat, (6) infraspinatus, (7) infraspinatus fat, (8) teres minor, (9) teres minor fat, (10) subscapularis, and (11) subscapularis fat. Only intramuscular fat—fat contained within the muscle border—was labeled for each muscle.

**Figure 4:**
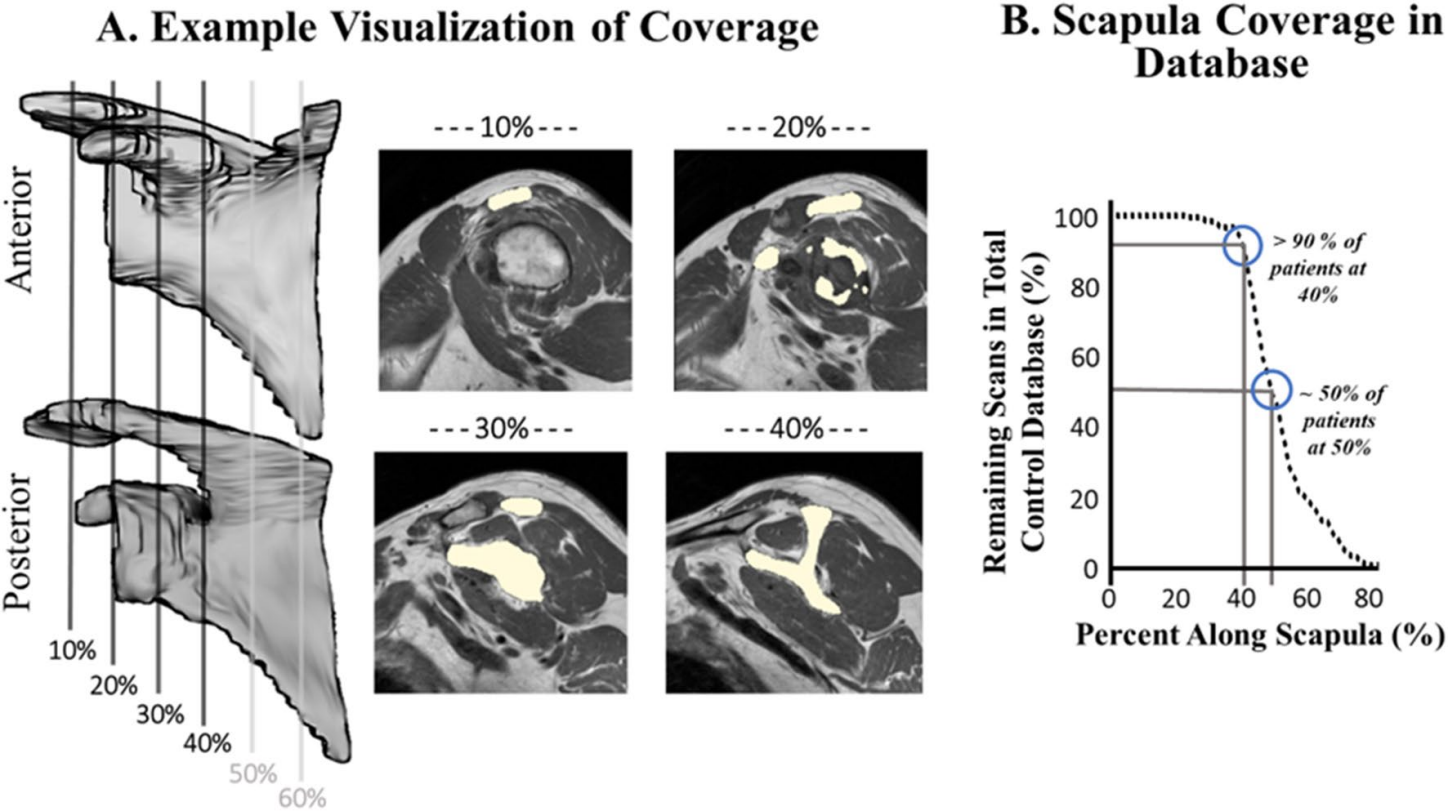
Demonstration of muscle morphology of the RC as a function of percent distance of scapula. (**A**) Anterior and posterior views of the coverage range as well as MRI slice examples (scapula labeled in white). (**B**) The number of scans from the control database (n = 170) that had image coverage at each percent of the scapula. Based on the 170 scans collected from the control database, approximately 90% of the scans captured at least 40% of the scapula, while less than 50% of the scans had 50% of the scapula captured.

The volume of each muscle RoI was expressed as a function of distance along scapula (Fig. 3C). To account for variation in scan coverage, the full scapula length is determined from the method described above (Fig. 3D) and then the volume of each muscle ROI was expressed as a function of the percent location along the scapula (Fig. 3E). The volumes of the muscle and scapula RoIs were interpolated between images and expressed as a function of percent distance into the scapula by 1% increments. Figure 4A provides a reference regarding what the scapula and rotator cuff musculature typically looks like as a function of percent distance along scapula, and Fig. 4B demonstrates the range in total coverage obtained in a cohort of clinical scans (n = 170).

Muscle size was defined as the muscle volume normalized for patient size by calculating the ratio of combined muscle and intramuscular fat volume to scapular volume where p is the percent location along the scapula:

1 $$Normalized\, Muscle \,Size\, (p)=\frac{\int\nolimits _{0}^{p}Muscle\, Area\, (p)\, dp}{\int\nolimits _{0}^{p}Scapula\, Area\, (p)\, dp},$$

Intramuscular fat was included in this calculation to measure the muscle volume and conform to methodology in previous literature^13^. Dividing muscle volume by scapula volume is commonly used to normalize by patient size when interpreting and comparing muscle volume across patients^13,16^. To validate this technique for partial volume measures, we correlated scapular volume and each of the four RC muscles’ volume as a function of location along scapula from 30 and 40% for the healthy adult scans. To avoid the potential effect of age, only adults < 40 years old were used for analysis. We also split the data between males and females to investigate the effect of sex on its relation to scapula normalization. This resulted in a total of 55 scans for this analysis, with at least 25 for each sex.

Relative contribution of each RC muscle was calculated for each muscle using the combined muscle volume (including intramuscular fat) compared to the total RC unit volume. This final ratio was then expressed as a percentage:

2 $$Relative\, Muscle\, Contribution\, (p)=\frac{\int\nolimits _{0}^{p}Muscle\, Area\,(p)\, dp}{\int\nolimits _{0}^{p}Total\, RC\, Unit\, Area\, (p)\, dp}\cdot 100,$$

Fatty infiltration was calculated as the ratio of intramuscular fat volume to the total muscle boundary volume:

3 $$Fatty\, Infiltration\, (p)=\frac{\int\nolimits _{0}^{p}Intramuscular\, Fat\, Area\, (p)\, dp}{\int\nolimits _{0}^{p}Muscle\, Area\, (p)\, dp}\cdot 100,$$

### Comparing partial to complete coverage RC musculature characteristics

Full coverage RC scans were used to examine the relationship between the total RC coverage and that at differing lateral coverages*.* Of the 47 complete coverage CT scans, 33 CT scans had complete coverage off all four RC muscles (the other 14 scans had clipping on the anterior/posterior portion of the muscles). Within the patient set used, 21 were preparing for a reverse total shoulder arthroplasty (likely torn/retracted RC), 3 for an anatomic total shoulder arthroplasty, and the remainder were preparing for surgery to correct for shoulder instability. This range in patient demographics was specifically chosen to further analyze if possible muscle injury and retraction could impact how regional muscle volumes are presented.

Segmentation of all four RC muscle boundaries (supraspinatus, infraspinatus, teres minor, and subscapularis) was performed by trained segmentation engineers on the CT scan for each patient. Using the same registration transformation from “Processing complete coverage CT and matched clinical MRI scans”, the segmentation was registered to the partial MRI scan to ensure a consistent slice orientation*.* Using the 3D segmentation of all four muscles and the scapula from the CT, three metrics were calculated: the total volume, normalized volume (muscle volume/scapula volume) and relative contribution (muscle volume/total of all four RC muscle volumes). As a comparison, these metrics were determined at 10%, 20%, 30% and 40% lateral coverage along the scapula’s length and correlated to the corresponding metric measured at total coverage.

### Effect of sex and age on RC characteristics

A two-way ANOVA was used to investigate the effect of sex (male, female) and age (15–29, 30–39, 40–49, 50–59, 60–69, 70–89 years) on each RC muscle’s size, relative contribution, and fat infiltration. Based on the work completed in this paper, the results were found using the values for the lateral 30% of scapula coverage because: (1) it was deemed reliable and (2) was most reflective of clinical MRI scans as most achieve at least 30% coverage. Of the 170 clinically obtained scans, 168 scans had at least 30% scapula coverage (Fig. 4B). Statistical analyses were performed using SPSS (Version 28, IBM, Chicago, IL, USA); Bonferroni corrections were used for multiple comparisons, and a significance level of alpha = 0.05 was used.

## Results

### Predicting scapula length from scapula lateral morphology

The multivariable linear regression showed that two variables were significantly correlated to the scapula length: sagittal distance of peak CSA (p < 0.001, t = 8.59) and peak CSA (p < 0.05, t = 2.24). By contrast, the vertical (p = 0.52, t = − 0.66) and horizontal (p = 0.781, t = − 0.28) bound were not significantly correlated to scapula length. The final regression’s relationship (r = 0.93, p < 0.001) for all variables was used to predict the scapula length in the 14 test scans. The average absolute error was found to be 2.92% with a standard deviation of 1.68%. The errors ranged from − 5.77 to 5.79%, illustrating no bias towards under- or over-predicting the scapula length.

### Comparing partial to complete coverage RC musculature characteristics

**Figure 5:**
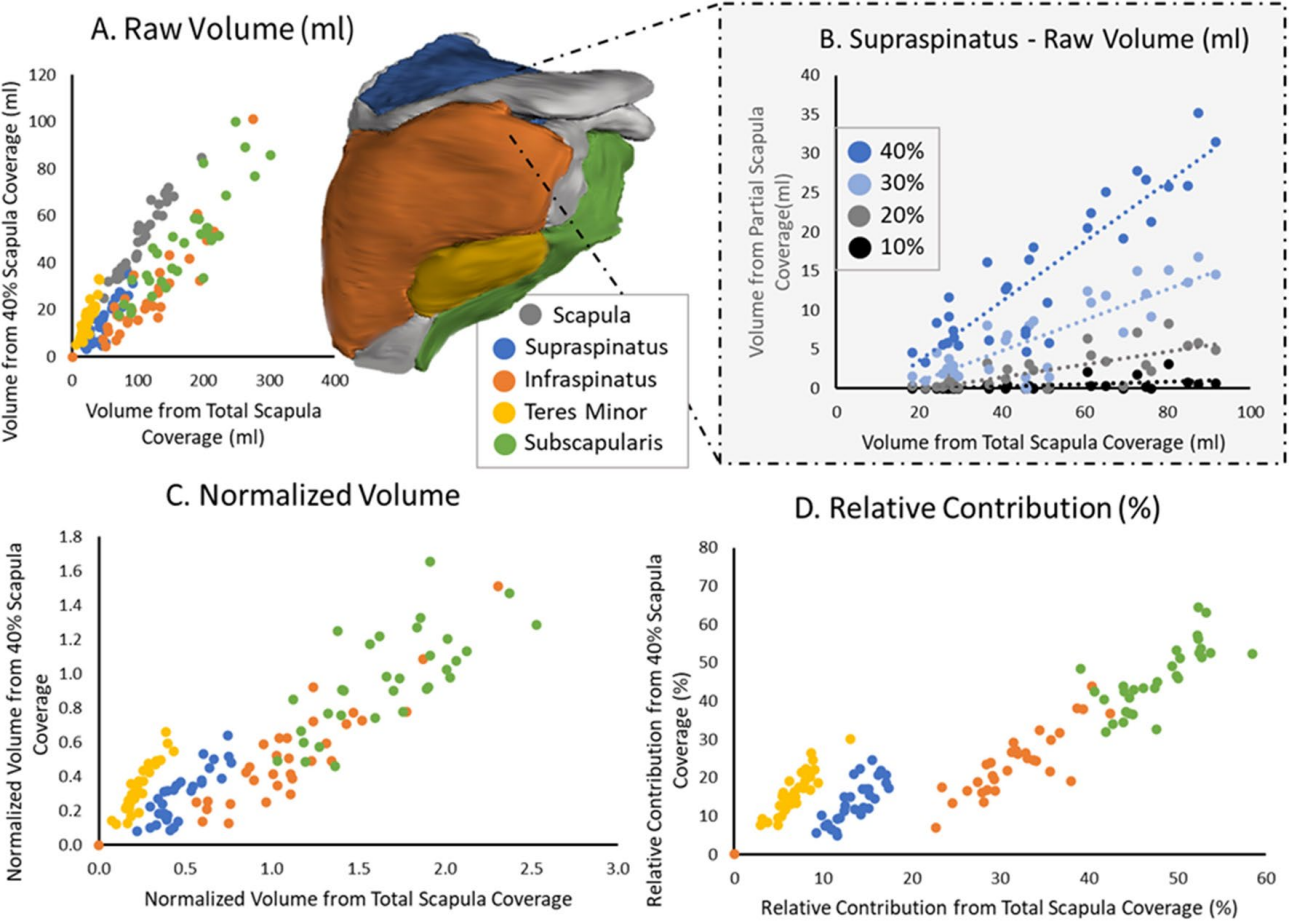
Correlation results for values found from partial lateral scapula coverages (10%, 20%, 30%, and 40%) as compared to values found from total coverage for the supraspinatus (blue), infraspinatus (orange), teres minor (yellow), and subscapularis (green) with example 3D volume rendering from an analyzed patient. (**A**) Raw volume (ml) correlation for all muscles from volume found at total coverage compared to that found at 40% lateral scapula coverage. (**B**) Raw volume (ml) correlation for the supraspinatus found at total coverage compared to that found at 10% (black), 20% (grey), 30% (light blue), and 40% (blue) lateral scapula coverage. (**C**) Normalized volume correlation for all muscles found at total coverage compared to that found at 40% lateral scapula coverage. (**D**) Relative contribution (%) correlation for all muscles found at total coverage compared to that found at 40% lateral scapula coverage.

When correlating raw volume, normalized volume, and relative contribution for all RC muscles obtained at varying lateral scapula coverages (10%, 20%, 30%, and 40%) as compared to values obtained from full coverage; consistently, coverage ranges of at least 30% achieved a strong significant correlation (Fig. 5 and Table 2). For raw volume, the scapula (r = 0.92), supraspinatus (r = 0.71), infraspinatus (r = 0.82), teres minor (r = 0.63), and subscapularis (r = 0.74) had high significant correlations starting at volumes measured at 30% lateral coverage to that measured at total coverage. The same pattern occurred for the normalized volumes and relative contribution in the supraspinatus (r = 0.82 and r = 0.66), infraspinatus (r = 0.82 and r = 0.72), teres minor (r = 0.82 and r = 0.79), and subscapularis (r = 0.57 and r = 0.63) at 30%. Correlations to total coverage tended to increase with lateral coverage.

**Table 2:** Correlation results for partial scapula coverages (10%, 20%, 30%, and 40%) as compared to total coverage results (raw volume, normalized volume, and relative contribution) for all RC muscles. A Pearson’s correlation was used if both datasets were normally distributed (using Shapiro wilks), otherwise Spearman’s correlation was used. *Signifies a significance < 0.05 was achieved.

Region	Raw volume (ml)				Normalized volume				Relative contribution (%)			
Scapula coverage	10%	20%	30%	40%	10%	20%	30%	40%	10%	20%	30%	40%
Scapula	0.65*	0.76*	0.92*	0.97*	–	–	–	–	–	–	–	–
Supraspinatus	0.58*	0.59*	0.71*	0.82*	0.58*	0.64*	0.82*	0.85*	0.00	0.55*	0.66*	0.79*
Infraspinatus	0.36*	0.67*	0.82*	0.88*	0.60*	0.74*	0.82*	0.85*	0.00	0.63*	0.72*	0.84*
Teres minor	0.00	0.33	0.63*	0.90*	0.38*	0.71*	0.82*	0.92*	0.00	0.57*	0.79*	0.90*
Subscapularis	0.09	0.44*	0.74*	0.87*	0.25*	0.30	0.57*	0.71*	0.00	0.38*	0.63*	0.80*

### Relationship between partial scapula and RC muscle volume as a function of scapula location

**Figure 6:**
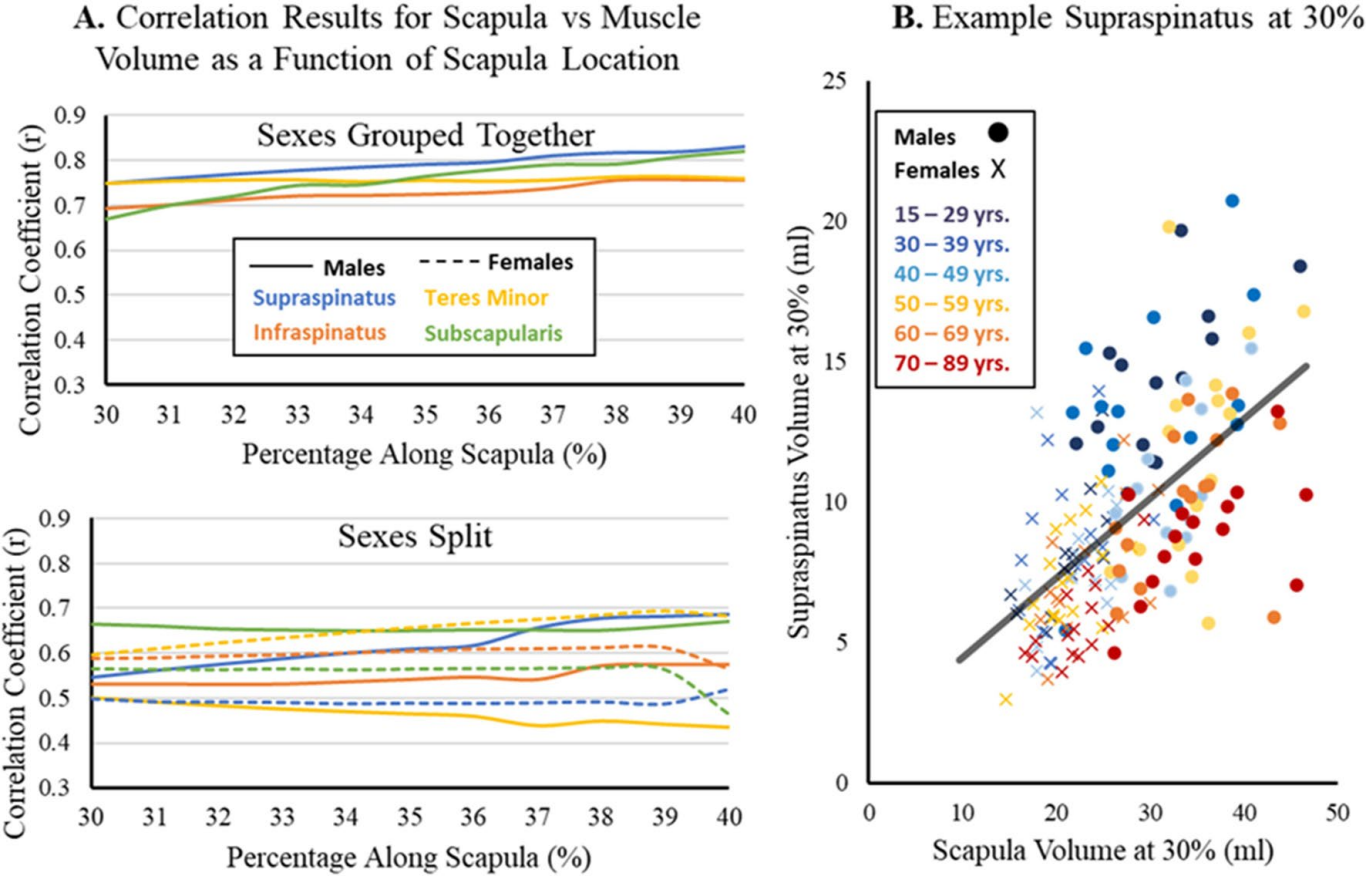
Relationship between partial scapula volume and rotator cuff muscle volume as a function of location along the scapula. (**A**) Correlation coefficient for the partial scapula volume related to the supraspinatus (blue), infraspinatus (orange), teres minor (yellow), and subscapularis (green) as a function of location along scapula (%) when analyzed as a group (top) and when split by sex (bottom) via males (solid) and females (dashed). A Pearson’s correlation was used if both datasets were normally distributed (using Shapiro wilks), otherwise Spearman’s correlation was used. All values had a p < 0.05. (**B**) Example correlation between the scapula and supraspinatus volume (ml) at 30%. Male participants are a circle symbol, while female participants are an x symbol. Patients between 15–29 years (dark blue), 30–39 years. (blue), 40–49 (light blue), 50–59 years. (yellow), 60–69 (orange), and 70–89 (red) are shown. While patients older than 40 where not used for analysis, they are shown here for visualization.

When relating scapula volume and RC muscle volume as a function of location along scapula (from 30 to 40%), all muscles demonstrated a strong and significant correlation (Fig. 6). Specifically, all muscles had a correlation coefficient of r > 0.65 (p < 0.001). The supraspinatus tended to have the strongest correlation and the infraspinatus the least. For all muscles, the correlation slightly increased from 30 to 40% along the scapula. When split by sex, males and females had very similar correlation coefficients (all p < 0.05); however, both sexes were slightly lower than the results of the sexes together.

### Effect of sex and age on RC characteristics

**Figure 7:**
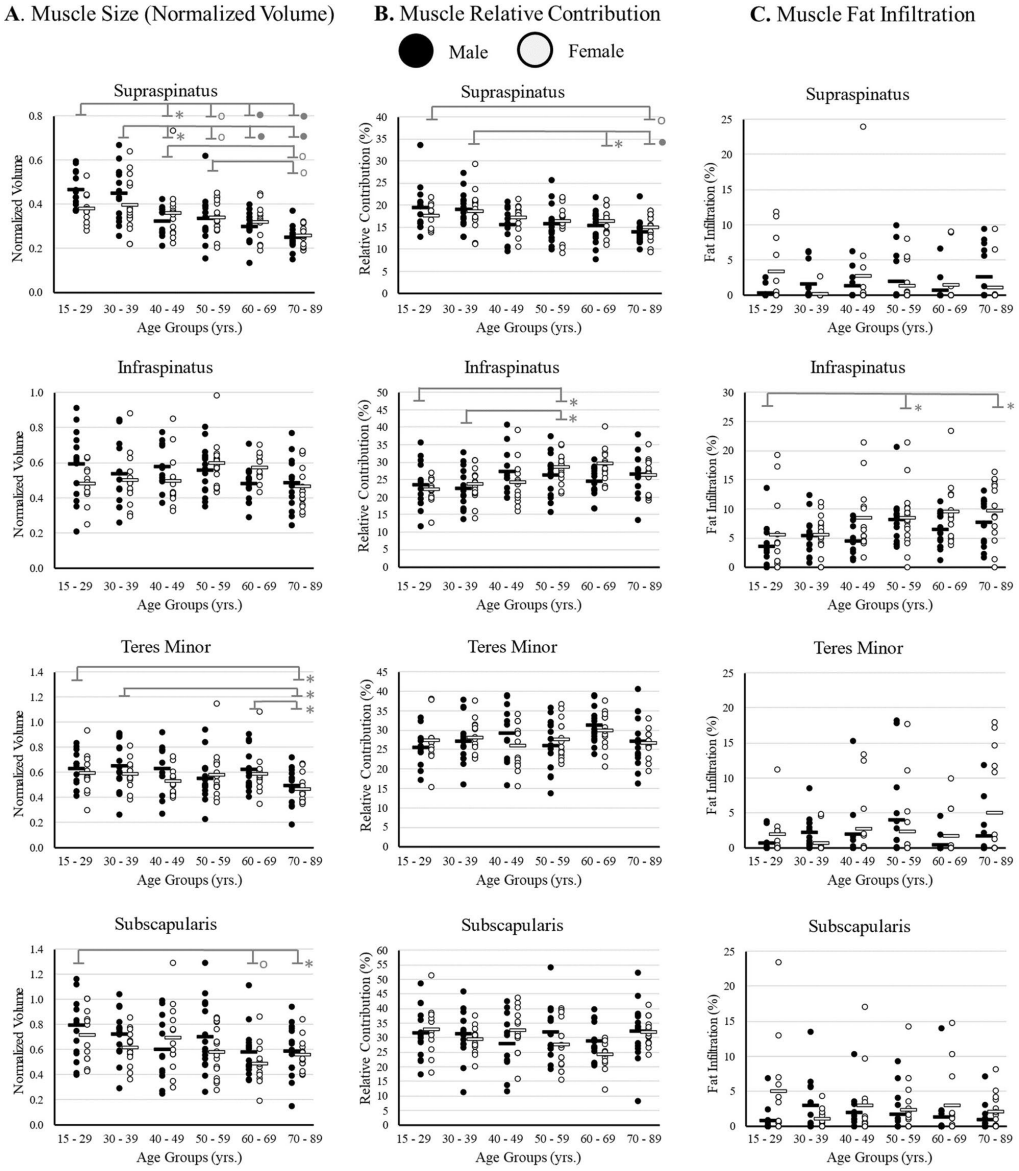
Analysis of the effects of age and sex on each rotator cuff muscle at 30% coverage for the metrics of (**A**) muscle size (normalized volume), (**B**) relative contribution (%), and (**C**) fat infiltration (%) for all six age subgroups for males (black) and females (light grey). Means are demonstrated by a horizontal line, and individual data points as dots. Shown is post-hoc analysis, in which * signifies a significance level of p < 0.05, ° is p < 0.01, and • is p < 0.001.

There was a trend of decreasing RC size with age for all muscles (Fig. 7A). Specifically, there was a significant main effect of age for the supraspinatus (F(5, 158) = 15.66, p < 0.001, partial η^2^ = 0.331), the teres minor (F(5, 158) = 3.13, p = 0.01, partial η^2^ = 0.09), and the subscapularis (F(5, 158) = 3.44, p < 0.01, partial η^2^ = 0.1).

With age, there was a trend in which relative contribution of the supraspinatus decreased while the infraspinatus increased (Fig. 7B). There was a significant main effect of age for the supraspinatus (F(5, 158) = 5.69, p < 0.001, partial η^2^ = 0.15) and the infraspinatus (F(5, 158) = 3.95, p < 0.01, partial η^2^ = 0.11).

For fat infiltration, the infraspinatus demonstrated an increase in fat infiltration with age, and in females (Fig. 7C). There was a significant main effect of sex for the infraspinatus (F(1, 158) = 8.18, p < 0.01, partial η^2^ = 0.05) and age (F(5, 158) = 3.91, p < 0.01, partial η^2^ = 0.11).

## Discussion

The goal of this work was to develop a novel approach to volumetric characterization of the RC muscle unit from limited-coverage clinical MR scans in order to allow for objective and holistic quantification of fatty infiltration and muscle volume and contribution. The characteristics of RC musculature have been documented in healthy adults^17^ and RC tear patients^13^ with complete MRI/CT scan coverage^18–20^. However, in clinical settings, scans of the RC have a limited medial capture range or image quality degrades as the scan moves medially. This study leveraged our previously developed AI-based segmentation method^14^ to examine the clinically captured RC musculature and fully leverage the available information via 3D analysis. Measurement of muscle volume and fatty infiltration of the constituent muscles of the RC unit as a function of capture range using scapular location in the sagittal plane is a novel approach to orthopedic care for RC tear patients that can allow for more holistic and objective clinical decision-making to improve patient outcomes.

### Normalization method for variation in scan range and patient size

This study employed novel normalization techniques that allowed for the utilization of existing data regardless of scan coverage or patient size. Total scapula length was predicted from morphological characteristics of the lateral scapula within a low (average of 3%) error, allowing for muscle analysis based on scapula location. Werthel et al. previously demonstrated the use of scapula normalization for muscle size estimates of full muscle captures^13^, which was reflected in the significantly strong relationships between muscle size and scapula volume as a function of scapula location observed in the results of this study. All correlation coefficients were greater than 0.65 and demonstrate the scapula’s strong ability to normalize muscle volume for patient size, without the concern of the effect of sex on normalization method. As discussed by Werthel, the advantage of using the scapula as a normalization standard for patient size is its consistency between patient populations and pathologies, providing a reliable benchmark.

### Partial RC musculature is predictive of complete coverage

When comparing fully captured RC musculature to that capped to lateral scapula coverage ranges of 10%, 20%, 30%, and 40%; RC musculature volume, normalized volume (size) and relative contribution found at 30% and 40% strongly and significantly correlated to that of total muscle coverage. This result aids in the proposed methodologies use, as the partially captured lateral region of the RC obtained from clinical MRIs can be used to make inferences of the total RC muscle’s size and relative contribution. Of note, the CT scans used were collected from a variety of patients, including those preparing to undergo shoulder replacement and patients with deemed healthy RC musculature. This aided in the interpretation that the relationship between partial coverage and full coverage is valid across a range of muscular pathologies. The total RC metrics found from the complete coverage CT match with that in current literature^13^, and further demonstrates the application of these findings to varying RC pathologies. However, a limitation of this approach is that tears, increased fatty infiltration, and other features are not captured in the medial 60% and cannot be predicted or foreseen. However, recent research has demonstrated that FI clustering likely occurs in the lateral regions of the muscles (supraspinatus, teres minor, and infraspinatus), especially in distal RC tears^10^. Of additional note, prior work has shown that along with proximal to distal gradation of fat infiltration from supraspinatus tendon detachment, it progresses with time^21^. By correcting for variation in scan coverage, progression can be assessed in multiple scans.

### Partial scan coverage and analysis determination

Through several validation steps, our work shown here demonstrates the ability to objectively estimate how much of a muscle is captured in partially acquired sagittal MRI scans as referenced to scapula location. When applied to a healthy adult cohort and used to examine the effect of age and sex on RC musculature characteristics, we used values at 30% coverage. The reasoning for this is twofold: (1) our validation in the paper demonstrates that results at 30% provide strong correlations to results captured at complete coverage and therefore is a reliable location to draw inferences, and (2) a majority of scans achieve at least 30% coverage (Fig. 4B), therefore conclusions drawn from this location would be more broadly applicable to a population of scans. Of note, if statistical analysis was run at 40% instead of 30%, the number of scans drops to 155 (with at least 10 in each subgroup). The same findings as in 30% are found; however, there is the addition of size of the infraspinatus significantly decreasing with age (main effect of age p < 0.05), relative contribution of the subscapularis decreasing with age (main effect of age p < 0.01), and females exhibiting higher fat infiltration levels of the subscapularis (main effect of sex p < 0.05). These were all trends that were close to significant at 30% but became significant at 40%.

### Effect of sex and age on RC characteristics

Our results demonstrate: (1) decreased size of the supraspinatus, teres minor, and subscapularis with age, (2) decreased supraspinatus and increased infraspinatus relative contribution with age, and (3) increased FI in the infraspinatus in females compared to males. Our results are consistent with previous literature, in which it was found that muscle atrophy is more affected by age than by pathology^11^, fat infiltration tends to increase with age^22^, and fat infiltration is higher in females in the subscapularis^23^ (our data demonstrated this as a trend). Of note, previous work has shown that RC muscles are smaller in females than males^23^; however, the study normalized muscle volume by body height in which other research found that male’s upper limb muscle volume scaled better with height and weight than females^17^, which could have biased the results. In this paper we investigated the effect of sex on scapula normalization and found that it wasn’t of concern. Work by Werthel normalized muscle volume by scapula volume in males and females separately, in which they found females normalized volume tended to be smaller than males^13,16^; however, they never directly investigated sex differences. Our results demonstrate similar findings, in which females tended to have lower normalized volumes than males; however, our work demonstrates that when controlling for age and utilizing a large dataset, laterally captured scapular normalized muscle volumes do not significantly differ between sexes.

This work can be further utilized to examine location specific patterns of fatty infiltration and muscle development. Work by Beeler et al. demonstrated that fatty infiltration may occur laterally from tendon retraction, and that differing fat infiltration shapes and localization may reflect RC tear or neuropathy^24^. Further, work by Davis et al. has demonstrated that differing fatty infiltration presentations are associated with RC tear pattern and size^25^. It is of note that while significant effects of fat infiltration on sex were found, these fat filtration levels are relatively low (< 10% on average) as to be expected within a healthy population. Therefore, while there may be a significantly higher levels of FI in females, this increased level is still relatively low. This study emphasizes the need for objectively measuring these features as a function of location along the scapula in clinical MRIs.

### Implementation of proposed method utilizing reference database

Current methods used to evaluate RC muscle atrophy are insufficient as they utilize a single image to obtain a 2D assessment of the musculature^8,9,26^. This subjective assessment does not properly capture the full muscle volume and fatty infiltration^27–29^, and usually focuses on the supraspinatus. In addition to the inability of standard assessments to accurately reflect the 3D musculature, 2D measurements often do not account for the expected muscle atrophy seen with aging. As an example, the tangent sign grades muscle atrophy in relation to the fossa^30^, and the Goutallier score grades fatty infiltration compared to muscle presence^6^. Therefore, muscle atrophy from aging can be falsely attributed to injury. We propose that the methodology validated here can be applied to patient populations (such as RC tears) to objectively quantify the RC size, relative contribution, and fatty infiltration. While in this paper, a cut off of the lateral 30% muscle coverage relative to the scapula was used to investigate sex and age effect, the work presented here demonstrates that muscle coverages from 30 to 40% can reasonably be used. This coverage range is captured in a majority of scans as well (168 out of the 170 in this paper), and therefore likely to be an applicable method for most clinical scans. By utilizing the correct reference database for a patient’s demographic as created here, a z-score can be used to relate the muscle characteristics and provide final volumetric scores. Additionally, with the use of AI to automatically segment the partial 3D RC structures from the clinical MRI, this proposed method is fast to implement. To be specific, it took 1 min to go from a clinical MRI scan to the AI segmentation of the RC musculature. As described in our previous paper, the AI is fairly accurate (~ 5% volume error, ~ 2% FI error) in both healthy adults and patients with RC tears^14^, however, corrections that may need to be made and vetting the segmentation can take about 5–10 min. Once the segmentations are finalized, the process to calculate the z-scores for all 4 muscles for the three different metrics is automatic and takes less than a minute per a scan. While this is indeed fast and clinically applicable, the only step that may cause an issue in a clinical setting is the need to correct the AI segmentation. Recent publications have shown techniques to automatically “grade” the AI segmentation’s accuracy in order to improve segmentation in the rotator cuff^31^. The ultimate goal is for the analysis to be used without the need for clinician vetting.

### Study limitations

As predicted, total scapula length was based on sagittal measures of the scapula’s lateral characteristics, it was investigated if variation in scapula orientation was common in MRI scans (which could impact scapula length measures), and it was found to be of minimal concern (Supplementary Material). The sample size of this research was large (n = 170), however, once split across differing age and sex groups, the subgroup sample size was a bit smaller (n > 10). While this was indicative of limited healthy clinical RC scans at the research site’s disposal, future work should expand and cover larger ranges of patient heterogeneity. For the results of this study, moderate to strong powers were achieved, signifying sample size is not as much of a concern for result interpretations.

Our study utilized percent along the scapula moving medially to correct for partial coverage, however, this may discount variation in muscle origin as seen in the teres minor (starting at the lateral scapula border) compared to the supraspinatus, infraspinatus, and subscapularis (medial scapula border start). While the partial to full volume validation from the CT scans demonstrates there is a strong correlation for the teres minor similar to that of the other three rotator cuff muscles, future work may improve the methodology to allow for better characterization of the partial teres minor volume utilizing other scapula characteristics.

A limitation of this study is that we did not apply this methodology to MRI scans of patients with pathologic shoulders. While using the CT scans to compare partial to full coverage results, we did not examine fat infiltration, only muscle volume. Patients with RC injuries may have differing distribution of fat infiltration. Our future work will examine the methodology’s application in patients with pathologic rotator cuff musculature. Specially, a point of possible concern arises as we transition this methodology in RC tear patients, by quantifying muscle volume as a function of location along the scapula, retraction could possibly influence the values. However, we argue in this paper that examining the amount of muscle present at specific points along the scapula is important, and therefore if retraction is present that will indeed influence the expected muscle present for that location and be still represented in the results (and interpreted as atrophy or retraction). Our future work will examine ways to create a “companion” measure of muscle retraction. We don’t believe this possible issue is of concern for immediate application in RC tear patients, as during our validation of partial compared to full muscle volume, some of the scans used were of patients with possible retraction and a strong correlation was still present. Further, one way to correct for this issue when applied in RC tear patient is to instead calculate muscle volume as a function of location along muscle (as normalized by location along scapula), and therefore retraction will not be a concern, and it is unlikely the results presented here in healthy adults would change as retraction should not be present. Finally, this study utilized clinical MRIs with typical, limited lateral coverage. The results of this research found at least 30% of the scapula should be captured to make accurate conclusions regarding RC musculature. While this could limit some scans from analysis, our work demonstrates that in a cohort of scans, 98% of scans will have at least 30% coverage.

## Conclusions

This study demonstrated a novel approach for utilizing clinically obtained shoulder MRI scans to analyze the entire 3D volume of all RC muscles and their respective fatty infiltration as a function of location along scapula. This method accounts for variation in scan coverage, patient size, and patient age as morphological characteristics of the lateral scapula can accurately predict total scapular length. When applied to a large cohort of healthy controls of varying age and sex, lateral characteristics of the RC musculature demonstrated diminished muscle size with age and increased fatty infiltration in females. Further, to assess how well partial 3D structures relate to their complete counterpart, RC muscle’s characteristics found in the lateral 30–40% of the scapula were correlated to those found with complete coverage, resulting in a strong, significant relationship. This methodology can be applied to patient populations to provide a z-score of expected muscle size, relative contribution, and fatty infiltration based on the control database. This provides an objective and comprehensive volumetric approach, that with automated AI segmentation and analysis, is fast and clinically attainable. The proposed method expands analysis to previously overlooked muscles and examines all available data collected during the clinical workflow, rather than a single image.

### Supplementary Information

Supplementary Information.

## Data Availability

Data generated or analyzed during the study are available from the corresponding author by request.

## Abbreviations

RC: Rotator cuff
MRI: Magnetic resonance imaging
CT: Computed tomography
3D: Three-dimensional
CSA: Cross sectional area
RoIs: Regions of interest
PD: Sagittal distance of peak CSA
PC: CSA at point
VB: Vertical bounding length of the scapula
HB: Horizontal bounding length of the scapula
