# Supplementary material for: Objective analysis of partial three-dimensional rotator cuff muscle volume and fat infiltration across ages and sex from clinical MRI scans
Source: Sci Rep. 2023 Sep 1;13:14345. doi: 10.1038/s41598-023-41599-z (PMC10474276; doi:10.1038/s41598-023-41599-z)
Supplement: Supplementary file 1 — Supplementary Information. [file 41598_2023_41599_MOESM1_ESM.docx]

**Full Article Title:** Objective Analysis of Partial Three-Dimensional rotator cuff muscle volume and fat infiltration across ages and sex from clinical MRI scans

**Authors:** Lara Riem PhD^1^*, Silvia S. Blemker PhD^1^, Olivia DuCharme ME^1^, Elizabeth B. Leitch MEd, ATC^2^, Matthew Cousins BS^1^, Ivan J. Antosh MD^3^, Mikalyn Defoor MD^3^, Andrew J. Sheean MD^3^, Brian C. Werner MD^2^

**Institutions:**

**^1^** Springbok Analytics, Charlottesville, VA, USA

**^2^** University of Virginia Medical School, Charlottesville, VA, USA

**^3^** San Antonio Military Medical Center, San Antonio, TX, USA

**Corresponding Author:**

Lara Riem

Phone: 608-434-1479

E-mail Address: lara.riem@springbokanalytics.com,

Address: 100 W. South Street, Suite 1E, Charlottesville, VA, USA 22902

**Supplemental Materials**

**Examining the Impact of Variation in MRI Scapula Orientation**

*Introduction*

To utilize partial volume captures of the RC muscles from clinically obtained retrospective sagittal MRI scans, the volume of each muscle as a function of location on the scapula moving medially was used. Therefore, to normalize and interpret results: 1) the volume of the RC muscles was represented as percentage along the scapula and 2) the slice-by-slice collection of these data points was collected along the scapular orientation. However, in clinically obtained scans the complete scapula is not captured. To predict total scapula length from distal characteristics of the scapula, complete captured CT scans and partial captured MRI scans were used. In order to use them together, they must be registered and therefore, sagittal measures must be taken in the same plane and orientation. A potential problem of this is that slight changes in the sagittal orientation of the scapula could lead to erroneous predictions of the scapula length. **The goal of this supplemental validation was to examine the typical variation in scapula orientation to discern if variation in scapula orientation could potentially be an error/confounding factor when predicting total scapula length from distal characteristics of the scapula**. It was found that the variation in MRI scapula orientation was low (average of less than 1 degree in the pitch orientation and < 4 degrees in the yaw orientation) and therefore unlikely to be a confounding factor.

*Dataset*

The dataset consisted of 47 patients with paired RC MRI and CT scans taken retrospectively (summary in **Table S1**) from a surgical clinic. The clinic was approved by an IRB (informed consent waived) and followed HIPAA compliance. All MRI scans were T1-weighted, acquired in the sagittal plane, and obtained during typical clinical workflow (not full RC coverage). All CT scans were full medial-lateral scapula coverage.

**Table S1**: Patient demographics for the paired MRI and CT RC scans. All MRI and CT scans were taken within 3 years of each other. Patients’ procedures were from anatomic and reverse total shoulder arthroplasties and latarjet procedures pre scans. Note for 9 of the scans used in the testing groups, a negligible amount of inferior scapula’ was omitted from the frame of view. While this did not impact the estimates used for scapula length, they were excluded from the orientation validation.

| **Age (yrs.)** | **Sex** | **RC Laterality** | **Scapula Length (cm)** |
| --- | --- | --- | --- |
| 44.80 ±22.97 | 18F, 29M | 19L, 28R | **Average**: 14.64 ± 1.72 cm  **Range**: 11.63 – 19.80 cm |

*Methods*

As noted, when comparing the partial MRI to the complete CT, orientation can be important for interpretation since the analysis is a function of sagittal location on scapula by slice. To investigate if this was an issue in sagittal clinical MRI scans, the variation in orientation was found. Below is a depiction of the scapula and coordinate system. Error could arise due to a rotation in pitch or yaw. Due to the difficult shape of the scapula, the registered full scapula CT to the MRI 3D rendering was utilized to find the orientation in 38 patients. To calculate the rotation, the 3D point in space that was directly in the center of the scapula’s label map bounds (COB) was found. A second point was found that was the 3D center of mass of the scapula label map (COM). The angle between these two points was used to define the rotation and variation in orientation (**Figure S1**).


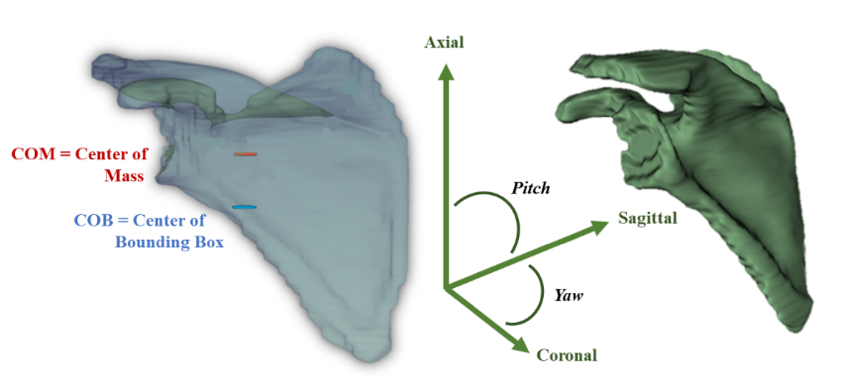


**Figure S1**: Example of how orientation of the scapula is found with respect to the sagittal plane. Shown is the 3D center of mass of the scapula label map (red, COM) and center of the scapula’s label map bounds (blue, COB).

*Results*

For the 38 patients the pitch rotation resulted in a mean and standard deviation of 0.83° ± 1.27°, and the yaw rotation was 3.94° ± 6.63°.

*Conclusions*

Given the low variation in pitch and yaw angle in the 38 clinical MRI examined here, it was deemed unlikely that variation in scapula orientation could be a confounding factor when estimating scapula length from lateral characteristics of the scapula.
